# Identification and evaluation of circulating exosomal miRNAs for the diagnosis of postmenopausal osteoporosis

**DOI:** 10.1186/s13018-023-04020-z

**Published:** 2023-07-26

**Authors:** Zhibang Sun, Junjie Shi, Chenyang Yang, Xukun Chen, Jiaqi Chu, Jing Chen, Yuan Wang, Chenxin Zhu, Jinze Xu, Guozhen Tang, Song Shao

**Affiliations:** 1 Department of Orthopedics, The Lu’an Affiliated Hospital of Anhui Medical University, Lu’an, People’s Republic of China; 2 Department of R&D, Echo Biotech Co., Ltd, Beijing, People’s Republic of China

**Keywords:** Plasma exosomes, MiRNA, Diagnostic efficacy, Postmenopausal osteoporosis, Number of pregnancies

## Abstract

**Background:**

Postmenopausal osteoporosis (PMOP) is a common condition that leads to a loss of bone density and an increased risk of fractures in women. Recent evidence suggests that exosomal miRNAs are involved in regulating bone development and osteogenesis. However, exosomal miRNAs as biomarkers for PMOP diagnosis have not been systematically evaluated. In this study, we aim to identify PMOP-associated circulating exosomal miRNAs and evaluate their diagnostic performance.

**Methods:**

We performed next-generation sequencing and bioinformatics analysis of plasma exosomal miRNAs from 12 PMOP patients and 12 non-osteoporosis controls to identify PMOP-associated exosomal miRNAs, and then validated them in an independent natural community cohort with 26 PMOP patients and 21 non-osteoporosis controls. Exosomes were isolated with the size exclusion chromatography method from the plasma of elder postmenopausal women. The plasma exosomal miRNA profiles were characterized in PMOP paired with controls with next-generation sequencing. Potential plasma exosomal miRNAs were validated by qRT-PCR in the validation cohort, and their performance in diagnosing PMOP was systematically evaluated with the receiver operating characteristic curve.

**Results:**

Twenty-seven miRNAs were identified as differentially expressed in PMOP versus controls in sequencing data, of which six exosomal miRNAs (miR-196-5p, miR-224-5p, miR320d, miR-34a-5p, miR-9-5p, and miR-98-5p) were confirmed to be differentially expressed in PMOP patients by qRT-PCR in the validation cohort. The three miRNAs combination (miR-34a-5p + miR-9-5p + miR-98-5p) demonstrated the best diagnostic performance, with an AUC = 0.734. In addition, the number of pregnancies was found to be an independent risk factor that can improve the performance of exosomal miRNAs in diagnosing PMOP.

**Conclusions:**

These results suggested that the plasma exosomal miRNAs had the potential to serve as noninvasive diagnostic biomarkers for PMOP.

**Supplementary Information:**

The online version contains supplementary material available at 10.1186/s13018-023-04020-z.

## Introduction

Osteoporosis accounts for more than 1.5 million fractures in the USA and 2.33 million fractures in China annually. Postmenopausal osteoporosis (PMOP) is the most common type of osteoporosis, characterized by decreased bone mineral density (BMD), the disintegration of bone microstructures, increased bone fragility, and easy fractures; it occurs in women after menopause as a result of increasing age and hormonal decrease [1]. As the global population ages, the incidence of PMOP rises dramatically, with about 50% of postmenopausal women worldwide affected by osteoporosis, and the prevalence of fractures in osteoporosis patients as high as 40% [2]. In 1994, a Working Group of the World Health Organization (WHO) established an operational definition of postmenopausal osteoporosis with BMD T score ≤ − 2.5 as the criteria [3]. Early detection and treatment of postmenopausal osteoporosis are essential to prevent fractures and maintain quality of life. The dual-energy X-ray absorptiometry (DXA) scan is the gold standard for diagnosing osteoporosis based on the measurement of bone mineral density (BMD) [4]. But the BMD usually show significant differences on an annual basis [5]. It is urgent to establish a more sensitive diagnosis method for osteoporosis.

Exosomes are small vesicles that are released by cells and contain a variety of biomolecules, including proteins, lipids, and RNA [6]. It participates in intercellular communication by delivering its inclusions [7]. Although different types of exosome components have been reported as markers for disease diagnosis, exosome miRNAs are the most widely reported as promising biomarkers for a range of diseases, including cancer, cardiovascular disease, and neurodegenerative diseases [8–12]. Compared with other components, exosomal miRNA is more abundant, and its detection method is more sensitive than protein [13]; compared to free miRNAs in the blood, exosomes can protect the miRNAs in them from degradation [10].

Previous in vitro and animal studies have shown that bone-marrow-derived mesenchymal stem cell (BMSC) derived exosomal miR-27a-3p, miR-196-5p, miR-29a, and miR-186 are involved in the mediation of bone angiogenesis and osteogenesis [14–16]. On the other hand, M1 macrophage-derived exosomes have been reported to aggravate bone loss in PMOP via a microRNA-98/DUSP1/JNK axis [17], and Mastocytosis-derived extracellular vesicles deliver miR-23a and miR-30a into pre-osteoblasts and prevent osteoblastogenesis and bone formation in the mouse model [18]. In addition, five plasma exosomal miRNAs, miR-224-3p, miR-25-5p, miR-302a-3p, miR-642a-3p, and miR-766-5p were identified as associated with PMOP by real-time PCR in the previous study [19]; mir-324-3p, mir-766-3p, mir-1247-5p, mir-330-5p, and mir-3124-5p have been reported to be associated with BMD in serum exosomes and may serve as potential diagnostic markers for PMOP [20]. These studies suggest that circulating exosomal miRNAs may be a promising diagnostic marker for PMOP; however, the systematic evaluation of circulating exosomal miRNAs as diagnostic markers of PMOP has not been fully investigated.

It is worth noting that in a study comparing the incidence of osteoporosis in North and South Korean women, the overall prevalence of osteoporosis was 48% (25/52) and 17% (27/156) among North Korean and South participants, while the North Korean subjects had a higher number of pregnancies[21]. Another study showed that breastfeeding was not associated with PMOP, but that the number of pregnancies increased the risk of PMOP in Chinese women [22]. This evidence suggests that the number of pregnancies may be a risk factor for PMOP.

In this study, we performed next-generation sequencing and bioinformatics analysis of plasma exosomal miRNAs from PMOP and non-osteoporosis controls, and systematically evaluated the diagnostic performance of PMOP-associated exosomal miRNAs in an independent cohort. Exosomes were isolated with the size exclusion chromatography (SEC) method from the plasma of elder postmenopausal women, whose average age was over 75 years. The plasma exosomal miRNA profiles were characterized in PMOP paired with controls with next-generation sequencing. Alterations of plasma exosomal miRNA were identified and compared with previous studies. Potential plasma exosomal miRNA markers were validated in a new independent validation cohort and their performance in diagnosing PMOP was systematically evaluated. In addition, we assessed the reliability of the number of pregnancies as a risk factor for PMOP. The purpose of this study was to identify plasma-derived exosomal miRNA biomarkers for PMOP diagnosis and evaluate their diagnostic performance.

## Materials and methods

### Plasma sample collection and exosome isolation

In the sequencing cohort, patients were older than 65 years with the BMD T score < − 2.5 and concurrent fractures. Healthy volunteers were over 65 years of age with the BMD T score > − 1 and no bone-related diseases (HIV and HBV infections were excluded). The validation cohort was composed of elderly women over 65 years of age from the natural community of Lu'an City, China (26 PMOP patients and 21 non-osteoporosis controls). This study was approved by the Ethics Committee of Lu’an People’s Hospital on December 28, 2020, with the approval number 2020LL-KY042. All patients and volunteers signed informed consent.

Peripheral blood samples from fasting individuals were collected in EDTA tubes following a regular venipuncture procedure. After centrifugation at 3000×*g* for 15 min at 4 °C, the plasma was aspirated and stored at − 80 °C before use. Dual-energy X-ray absorptiometry (GE Healthcare, USA) was performed to evaluate the bone mineral density (BMD) for subjects, and T score < − 2.5 reflected osteoporosis according to World Health Organization criteria [4].

The exosomes were isolated by SEC (size exclusion chromatography) methods as described previously with minor modifications [23]. Each 1-mL plasma was filtered by 0.8-μm PES material filter membrane (produced by Jinteng) and was diluted with 1.5-fold PBS, and it was further purified using Exosupur^®^ columns (Echobiotech, China) according to the manufacturer’s instructions. Then, the exosomal fractions were concentrated to 200 μL by 100 kDa molecular weight cut-off Amicon^®^ Ultra spin filters (Merck, Germany).

### Nano-flow cytometer

The size distribution and particle concentration of EVs were measured by using the nano-flow cytometer (N30E Nanoflow Analyzer, NanoFCM). Briefly, the side scatters intensity (SSI) was detected by the loading of the standard polystyrene nanoparticles (250 nm) to the nano-flow cytometer. Next, the isolated exosomes sample diluted with PBS (according to BCA Protein Assay results diluted the exosomes to 1–10 ng/μL) was loaded into the nano-flow to measure the SSI. Finally, the concentration of exosomes was calculated according to the ratio of SSI to particle concentration in the standard polystyrene nanoparticles. The size distribution was calculated according to the standard curve, which was generated by standard silica nanoparticles with mixed size (68 nm, 91 nm, 113 nm, 155 nm).

### Transmission electron microscopy (TEM)

Exosomes solution with 10 µL was placed on a copper mesh at room temperature for 10 min. After washing with sterile distilled water, the exosome was contrasted by uranyl acetate solution for 1 min. The sample was then dried for 2 min under incandescent light. The copper mesh was observed and photographed under a transmission electron microscope (H-7650, Hitachi Ltd., Tokyo, Japan).

### Western blot analysis

The exosomes supernatant was denatured in 5 × sodium dodecyl sulfonate (SDS) buffer and subjected to western blot analysis (10% SDS–polyacrylamide gel electrophoresis; 50 µg protein/lane) using rabbit polyclonal antibody CD9 (60,232-I-Ig, Proteintech, Rosemont, IL, 1.50 mg/mL), HSP70 (ab181606, Abcam, USA, 1.12 mg/mL), Alix (ab186429, Abcam, USA, 1.25 mg/mL), and Calnexin (ab22595, Abcam, USA, 1 mg/mL). The PVDF membranes were used and blocked with 5% skim milk powder. The antibodies and secondary antibodies were diluted with NCM Universal Antibody Diluent (Cat.No: WB500D) at 1:1000 and 1:5000, respectively. The secondary antibodies were the rabbit second antibody from Proteintech (Cat.No. SA00001-2).

### Exosomal RNA extraction, library preparation, and Sequencing

Total RNA was extracted from plasma exosomes using miRNeasy Serum/Plasma Advanced Kit (Qiagen, cat. No. 217204) according to the kit instruction. Exosomes (200 µL) are mixed with 60 µL Buffer RPL to release and stabilize RNA. The sample is then mixed with 20 µL Buffer RPP and centrifuged to precipitate proteins. Isopropanol is added to the supernatant to provide the appropriate conditions for RNA molecules to bind to the silica membrane. The sample is then applied to the RNeasy UCP MinElute spin column, where RNA binds to the membrane and other contaminants are washed away in subsequent wash steps. In the final step, the total RNA is eluted using RNase-free water.

Small RNA libraries were generated using the QIAseq miRNA Library Kit (Qiagen, Frederick, MD) following the manufacturer’s recommendations. In an unbiased reaction, adapters are ligated sequentially to the 3′ and 5′ ends of 5 µL total RNA on ice; the ligated RNA was reverse-transcribed into cDNA using a reverse transcription (RT) primer with Unique Molecular Index (UMI). Subsequently, the cDNA was cleaned with QIAseq Beads, and Library amplification was performed with a universal forward primer and indexing reverse primers. Then, the library quality was assessed on the Agilent Bioanalyzer 2100. Finally**,** the libraries were sequenced on an Illumina NovaSeq 6000 platform with 2 × 150 bp paired-end sequencing in an S4 flow cell at EchoBiotech Co. Ltd., Beijing, China.

### Quantification and differential expression analysis of exosomal miRNA

The Bowtie software was used to align Clean Reads with the Silva database, GtRNAdb database, Rfam database, and Repbase database, respectively. Then, the repeats and ncRNAs, such as ribosomal RNA (rRNA), transfer RNA (tRNA), small nuclear RNA (snRNA), and small nucleolar RNA (snoRNA), were filtered.

The remaining reads were used to detect miRNAs by comparing them with known miRNAs from miRbase and Human Genome (GRCh38), respectively. The expression matrix of quantified UMI counts of miRNAs was normalized to transcript per million (TPM). Differential expression analysis of two groups was performed using the edgeR package (3.12.1) [24]. *p* < 0.05 was used as the parameter to select differentially expressed miRNAs (DEMs).

### GO and KEGG pathway enrichment analysis

The target genes of DEMs were predicted by miRanda and RNAhybrid [25, 26]. Then, the target genes were aligned to Gene Ontology (GO) and the Kyoto Encyclopedia of Genes and Genomes (KEGG) database and confirmed their potential biological functions. KEGG pathway enrichment was analyzed using a Python program KOBAS [27]. GOseq R package based on Wallenius non-central hypergeometric distribution was used for GO enrichment analysis.

### Verification of candidate miRNAs with qRT-PCR

The method of exosomal RNA extraction is consistent with the above section. The total RNA was then reverse-transcribed to synthesize cDNA using PrimeScript™ RT reagent Kit (Perfect Real Time) (TAKARA, RR037A). The abundance of target gene expression was detected by the TaqMan^®^ probe using real-time qPCR (TaKaRa Ex Taq Hot Start Version, Takara, RR006A)). The 2 µL of cDNA was used as the template for each PCR reaction. Three technical replicates were performed for each qPCR reaction. The sequence of primers and probes are shown in Additional file 1. U6 was used as an internal reference, and the relative expression level of miRNA was calculated with 2^−ΔΔct^.

### Statistical analysis

All statistical analyses were calculated by GraphPad Prism version 9.0. For pair-wise comparisons, parametric *t* test and nonparametric Mann–Whitney test were applied based on the data distribution (the number of pregnancies was tested by a nonparametric test); *p* < 0.05 was considered statistically significant. The receiver operating characteristic (ROC) curve was used to evaluate the diagnostic efficiencies of the exosomal miRNA, and logistic regression was applied to determine the diagnostic performance of the combinations. The Pearson correlation coefficient was calculated for correlation analysis.

## Results

### Characterization of plasma exosomes

**Fig. 1:**
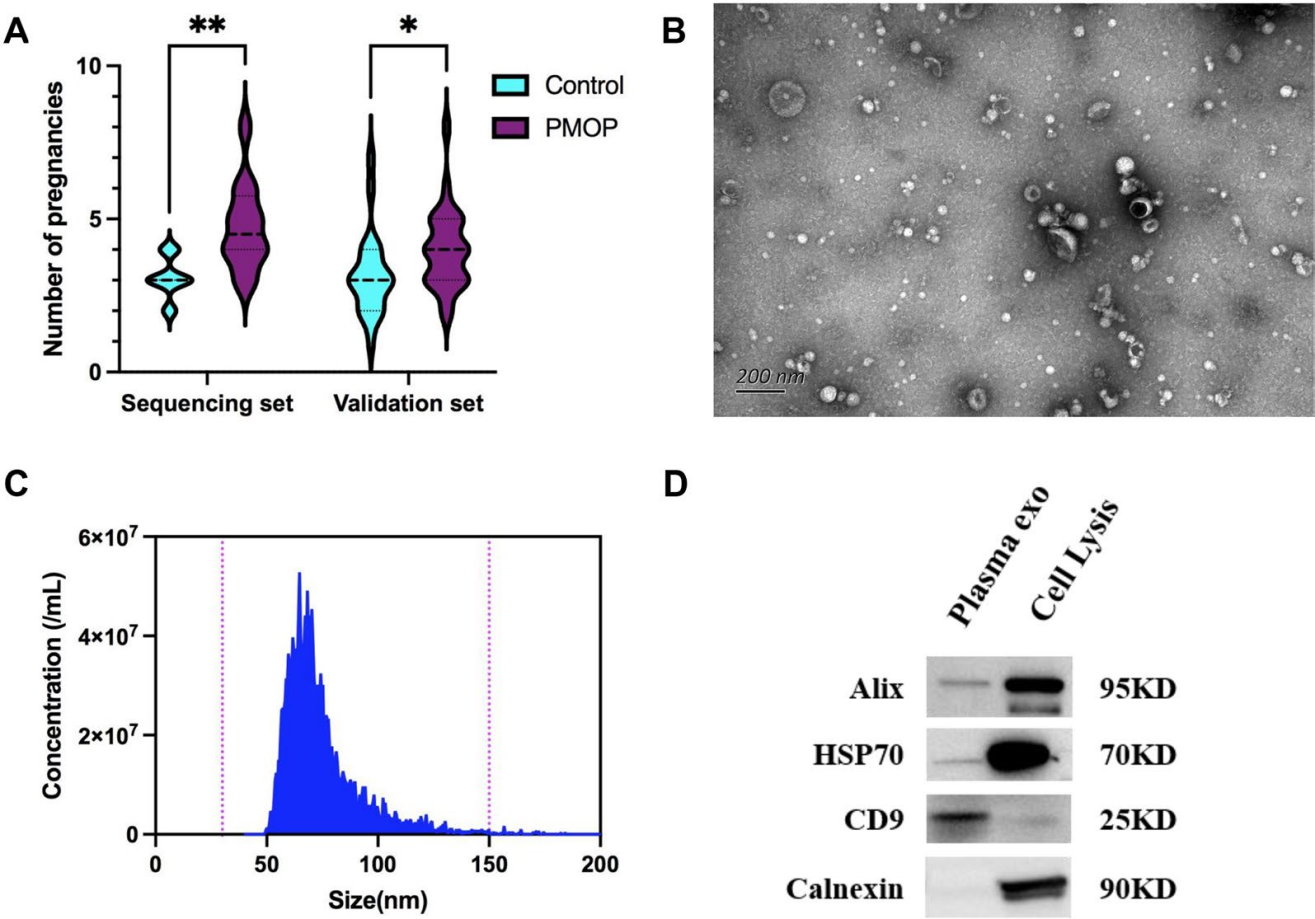
Identification of Plasma exosomes by SEC. **A** The comparison of the number of pregnancies between PMOP and control group in the sequencing set (*p* = 0.0013) and validation set (*p* = 0.0433), the number of pregnancies was tested by nonparametric Mann–Whitney test. **B** TEM image of the plasma exosomes which were cup-shaped vesicles, Scale bar on the left represents 200 nm. **C** NanoFCM results showed that plasma exosomes purified by SEC were enriched at 50–100 nm. **D** Western Blot for plasma exosomes, exosome-specific marker CD9, HSP70, and Alix were detected in plasma exosomes and the negative marker Calnexin was absent in the exosomes sample. The cell lysate here is derived from HEK293 cells, which was taken as the positive control

In this study, the sequencing cohort included 12 patients with osteoporosis and accompanying fractures, while the control group consisted of 12 healthy individuals of the same age with normal bone density. The validation cohort was composed of elderly women over 65 years of age from the natural community, which consisted of 26 PMOP patients and 21 non-osteoporosis controls. The clinical information on age, height, BMD, weight, number of pregnancies, and menopause age is summarized in Table 1. We found significant differences in the number of pregnancies in both cohorts (*p* = 0.0013 in the sequencing set, *p* = 0.016 in the validation set, (Fig. 1A), and the detailed data were listed in Additional file 2), suggesting that the number of pregnancies may be a risk factor for PMOP.

**Table 1:** Clinical characteristics of enrolled samples

	Sequencing set			Validation set		
	Control	PMOP	*p* value	Control	PMOP	*p* value
Sample	12	12		21	26	
Age (years)	78.67 ± 3.70	80.75 ± 4.50	NS	75.47 ± 6.58	77.04 ± 11.77	NS
Height (cm)	156.42 ± 3.77	159.08 ± 2.06	NS	158.9 ± 4.08	156.69 ± 21.84	NS
Weight (Kg)	60.24 ± 9.24	60.33 ± 13.55	NS	58.23 ± 8.14	55.57 ± 10.39	NS
BMD (g/cm2)	− 0.12 ± 0.48	− 3.52 ± 0.46	< 0.000001	− 1.29 ± 1.03	− 3.54 ± 0.82	< 0.000001
Number of pregnancies	3.08 ± 0.64	4.75 ± 1.36	0.0013	3.19 ± 1.33	4.00 ± 1.36	0.016
Menopause age (years)	48.67 ± 5.28	48.83 ± 4.22	NS	50.61 ± 3.01	48.80 ± 7.80	NS
Fractures	12/12	0/12		3/21	5/26	
Diabetes	0/12	0/12		4/21	6/26

The exosomes were isolated from plasma by size exclusion chromatography, and the morphology and size distribution of exosomes were characterized by TEM and NanoFCM as recommended in MISEV2018 guidelines [6]. TEM and NanoFCM results showed that the plasma exosomes were cup-shaped with a size range between 50 and 150 nm (Fig. 1B, C). Exosome-specific protein markers CD9, HSP70, and Alix were detected in plasma exosomes by western blot, while the negative marker Calnexin was not present in the exosomes sample (Fig. 1D). These characterization data indicate that our isolated plasma exosomes are typical, laying the foundation for their further research.

### miRNA sequencing and differential miRNA identification in PMOP plasma exosomes

**Fig. 2:**
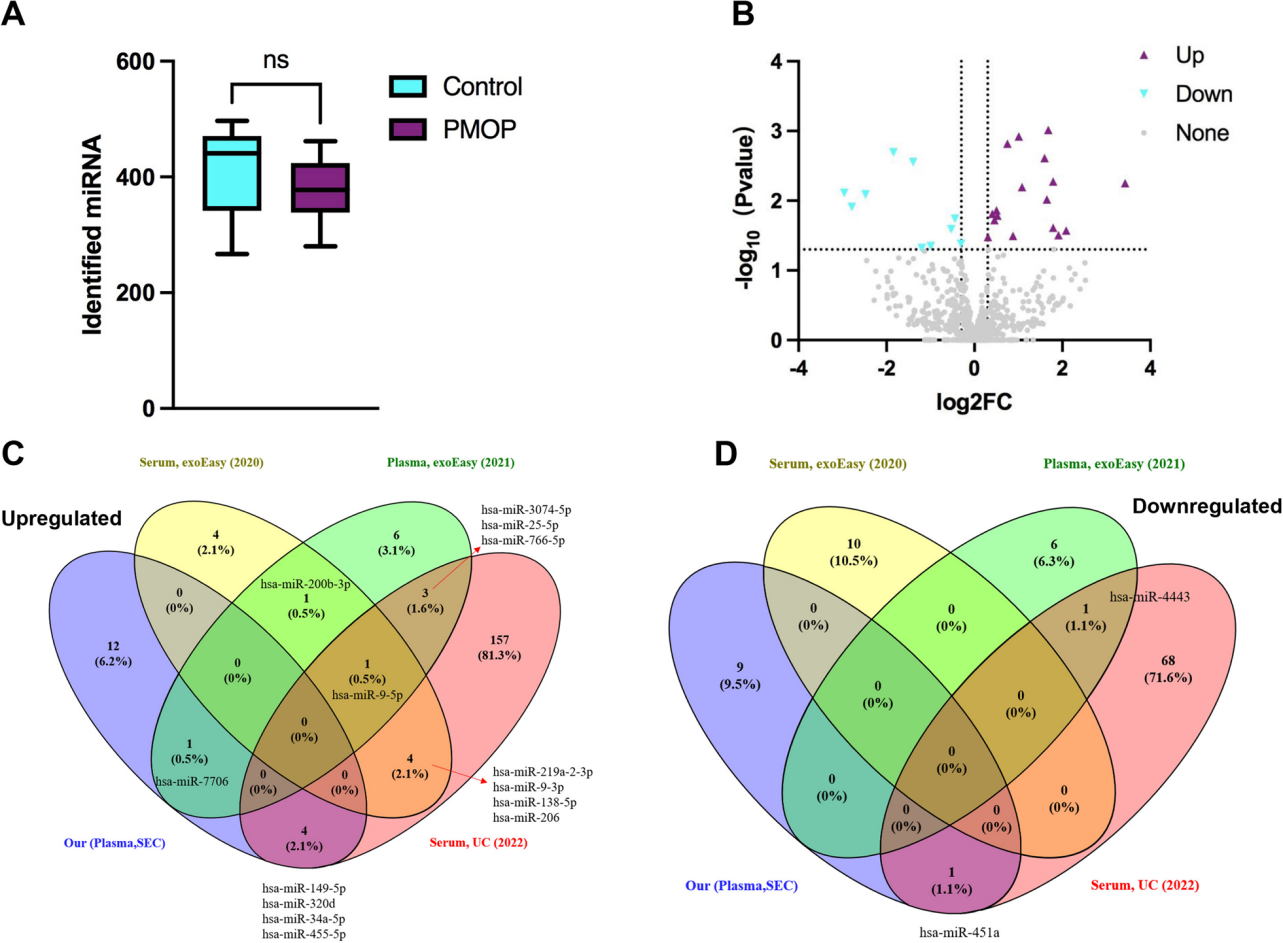
miRNA sequencing and differential miRNA identification in PMOP plasma exosomes. **A** Identified miRNA number in PMOP and control group, the comparisons of the two groups were based on an unpaired Student’s *t* test, ns indicates that there is no significant difference between the two groups (*p* > 0.05). **B** Differentially expressed miRNA volcano plot, where purple dots represent miRNAs upregulated in PMOP over control and blue dots represent down-regulated miRNAs. **C** Venn plots for comparing upregulated miRNAs to three other published datasets. **D** Venn plots for comparing down-regulated miRNAs to three other published datasets

To study the miRNA profiles of PMOP plasma exosomes, we generated miRNA sequencing of the sequencing cohorts (12 PMOP patients, 12 control). After removing the low-quality reads and the sequences smaller than 18 nt or longer than 32 nt, 24 samples had an average of 9.15 M clean reads with each sample from 6.21 M to 14.82 M (Additional file 3: Fig. S1), which were higher than the minimum saturated reads (2.50 M) of plasma exosome miRNA sequencing reported in the previous study [28]. There was no significant difference in clean reads between PMOP and the control group (*p* = 0.7179). A total of 984 known miRNAs were identified in all samples. Among them, the average number of miRNAs identified in the PMOP group was 376, and the control group was 412 with no significant difference between the two groups (*p* = 0.1841) (Fig. 2A).

The results of differential expression miRNA (DEMs) analysis showed that 17 miRNAs were significantly upregulated, and ten miRNAs were significantly down-regulated in PMOP patients than the control group (Fig. 2B, Table 2). Three published DEM datasets were incorporated for cross-validation (Additional file 4), which examined the exosomal miRNAs expression differences in osteoporosis patients by miRNA sequencing and used different sample sources or isolation methods: 1. Serum exosomes purified by exoEasy [29]; 2. Plasma exosomes isolated by exoEasy [19]; 3. Serum exosomes were separated by UC [20]. Five of the 17 upregulated miRNAs appeared in the other three datasets, including miR-34a-5p, miR-149-5p, miR-320d, miR-455-5p, and miR-7706 (Fig. 2C). And one of the ten downregulated miRNAs intersects with the three datasets, which is miR-451a (Fig. 2D). Additionally, eight upregulated miRNAs and one down-regulated miRNA appeared twice in the other three datasets, and one upregulated miRNA (miR-9-5p) appeared in all three datasets. Approximately, 22% (6/27) of the DEMs were reported in previous studies, indicating the reliability of our study. It also clarifies that more new PMOP-related exosomal miRNAs have been discovered in this study.

**Table 2:** Differential expression exosomal miRNA between PMOP with control group

No	Upregulated			Down-regulated		
	miRNA	*p* value	log2FC	miRNA	*p* value	log2FC
1	hsa-miR-196a-5p	0.0010	1.6773	hsa-miR-494-3p	0.0478	− 1.1970
2	hsa-miR-224-5p	0.0012	1.0100	hsa-miR-381-3p	0.0450	− 0.9910
3	hsa-miR-34a-5p	0.0015	0.7524	hsa-miR-425-5p	0.0416	− 0.3053
4	hsa-miR-205-5p	0.0024	1.5941	hsa-miR-484	0.0255	− 0.5283
5	hsa-miR-375-3p	0.0053	1.7906	hsa-miR-451a	0.0181	− 0.4449
6	hsa-miR-149-5p	0.0056	3.4252	hsa-miR-377-3p	0.0122	− 2.7876
7	hsa-miR-335-3p	0.0064	1.0849	hsa-miR-132-5p	0.0081	− 2.4780
8	hsa-miR-6724-5p	0.0096	1.6498	hsa-miR-106a-3p	0.0077	− 2.9616
9	hsa-miR-320c	0.0137	0.5010	hsa-miR-369-5p	0.0028	− 1.3939
10	hsa-miR-152-3p	0.0154	0.4070	hsa-miR-542-5p	0.0020	− 1.8426
11	hsa-miR-664a-5p	0.0164	0.5245			
12	hsa-miR-320d	0.0190	0.4566			
13	hsa-miR-455-5p	0.0244	1.7881			
14	hsa-miR-7706	0.0268	2.0883			
15	hsa-miR-1299	0.0313	1.9126			
16	hsa-miR-190b-5p	0.0319	0.8762			
17	hsa-miR-98-5p	0.0333	0.3047			

**Fig. 3:**
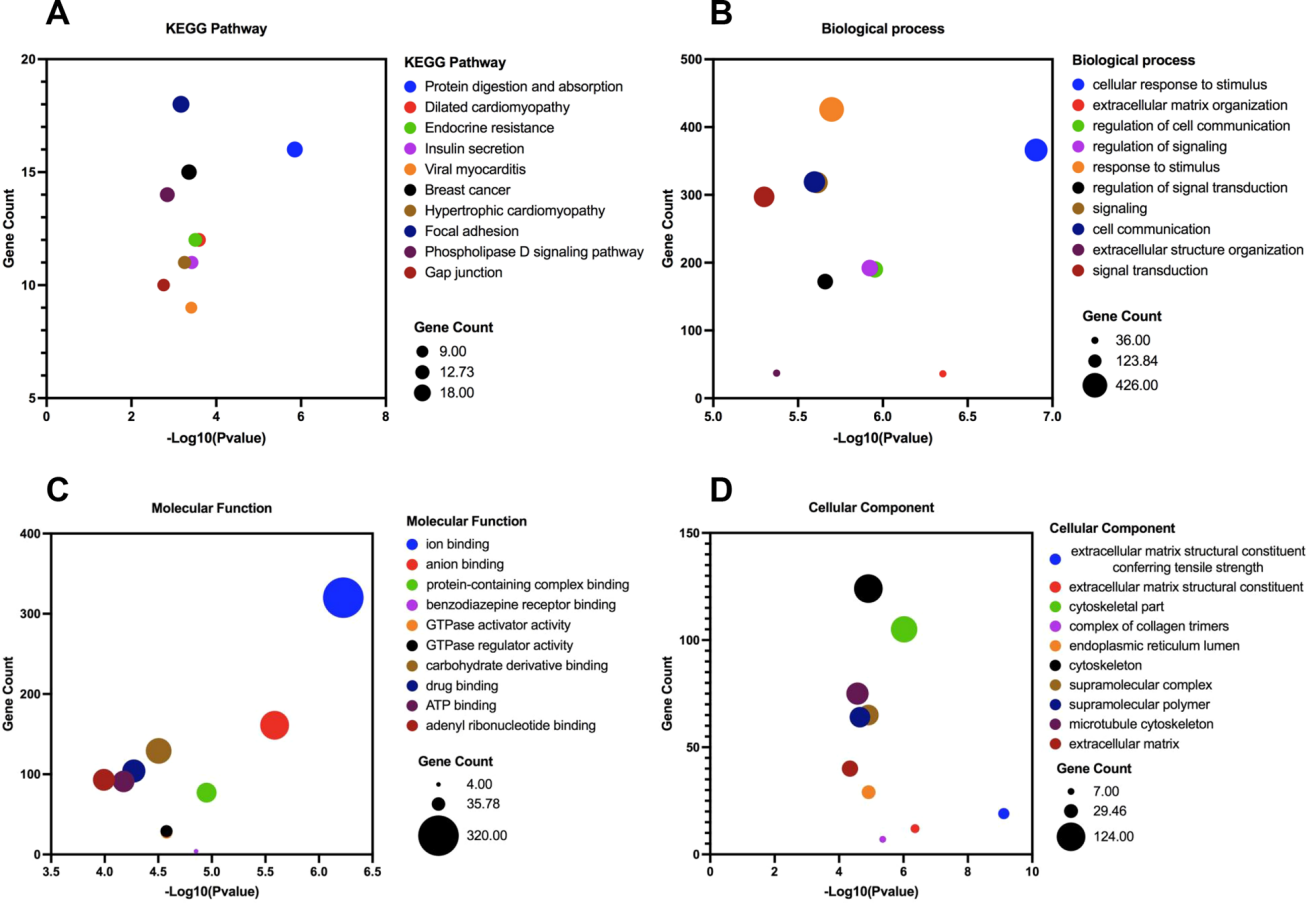
KEGG and GO enrichment analysis of target genes for differential exosomal miRNAs in sequencing data. **A** A bubble plot of the top 10 enriched KEGG pathways. **B** A bubble plot of the top 10 enriched GOs (Biological Process). **C** A bubble plot of the top 10 enriched GOs (Molecular Function). **D** A bubble plot of the top 10 enriched GOs (Cellular Component)

Based on bioinformatics database analysis, these 27 DEMs may target a total of 919 coding genes (Additional file 5). The top 10 significant pathways in the KEGG enrichment analysis of target genes are found in Fig. 3A, and the most relevant pathways were *protein digestion and absorption*, *dilated cardiomyopathy*, *endocrine resistance*, and *insulin secretion*. The biological process (BP), molecular function (MF), and cellular component (CC) in the GO enrichment analysis are shown in Fig. 3B–D. *Cellular response to stimulus*, *extracellular matrix organization* and *regulation of cell communication* in BP, *ion binding*, *anion binding*, and *protein-containing complex binding* in MF, *collagen trimer*, *extracellular matrix component*, and *cytoskeletal part* in CC were the most significant enrichment GO terms.

### Validation of PMOP-related exosomal miRNA

Our 27 DEMs and ten miRNAs that intersect in the other three datasets in the above section made a total of 37 miRNAs, which were then filtered with the median TPM > 50 both in the PMOP and control group, leaving 11 candidate miRNAs for subsequent validation. Considering that miR-9-5p was present in three datasets, it was also included in the final validation (Table 3).

**Table 3:** Candidate exosomal miRNAs for RT-qPCR verification

miRNA	Sequencing set				Data set	Validation set	
	Median expression control	Median expression PMOP	*p* value	Change		*p* value	Change
hsa-miR-152-3p	94.05	126.98	0.0154	Up	1	0.0650	None
hsa-miR-196a-5p	117.04	142.18	0.0010	Up	1	0.0297	Up
hsa-miR-224-5p	86.86	129.64	0.0012	Up	1	0.0179	Up
hsa-miR-320c	159.11	215.77	0.0137	Up	1	0.8828	None
hsa-miR-320d	101.28	130.05	0.0190	Up	2	0.0292	Up
hsa-miR-34a-5p	53.36	87.25	0.0015	Up	2	0.0397	Up
hsa-miR-98-5p	364.00	404.79	0.0333	Up	1	0.0377	Up
hsa-miR-425-5p	1079.99	842.75	0.0416	Down	1	0.0970	None
hsa-miR-451a	4473.88	3378.84	0.0181	Down	2	0.9880	None
hsa-miR-484	116.33	96.65	0.0255	Down	1	0.2221	None
hsa-miR-200b-3p	202.02	196.69	0.3049	None	2	0.0790	None
hsa-miR-9-5p	14.78	19.65	0.5705	None	3	0.0193	Up

**Fig. 4:**
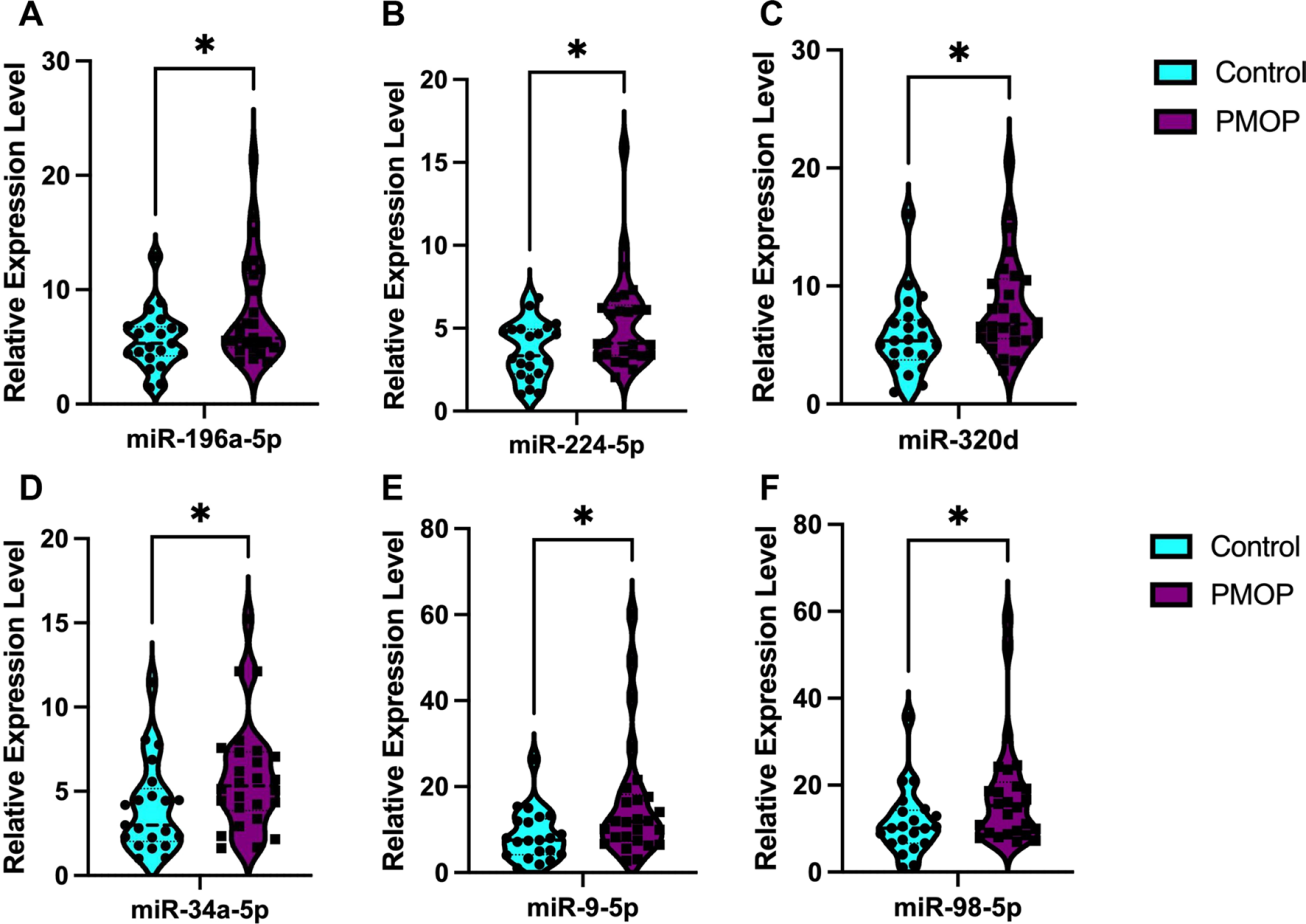
Relative expression levels (calculated by 2^−ΔΔct^) of plasma exosomal miRNA candidates in the validation set. The comparisons of the two groups were based on an unpaired Student’s *t*-test, with *p* < 0.05 as the significance threshold. **A** miR-196a-5p, **B** miR-224-5p, **(C)** miR-320d, **D** miR-34-5p, **E** miR-9-5p, **F** miR-98-5p. *means *p* < 0.05

To confirm these 12 PMOP-associated exosomal miRNAs, the expression of these miRNAs was measured by RT-qPCR in an independent cohort of 47 individuals (26 PMOP samples and 21 control samples). MiR-196-5p, miR-224-5p, miR320d, miR-34a-5p, miR-9-5p, and miR-98-5p were shown to be significantly upregulated in PMOP than control groups, and all of them were consistent with our sequencing data or previously reported (miR-9-5p) (Fig. 4, Table 3). There were no significant differences of miR-152-3p, miR-200b-3p, miR320c, miR-425-5p, miR-451a, and miR-484 in the validation cohorts (Table 3, Additional file 3: Fig. S2).

### Evaluation of the diagnostic efficacy of the validated exosomal miRNA for PMOP

**Fig. 5:**
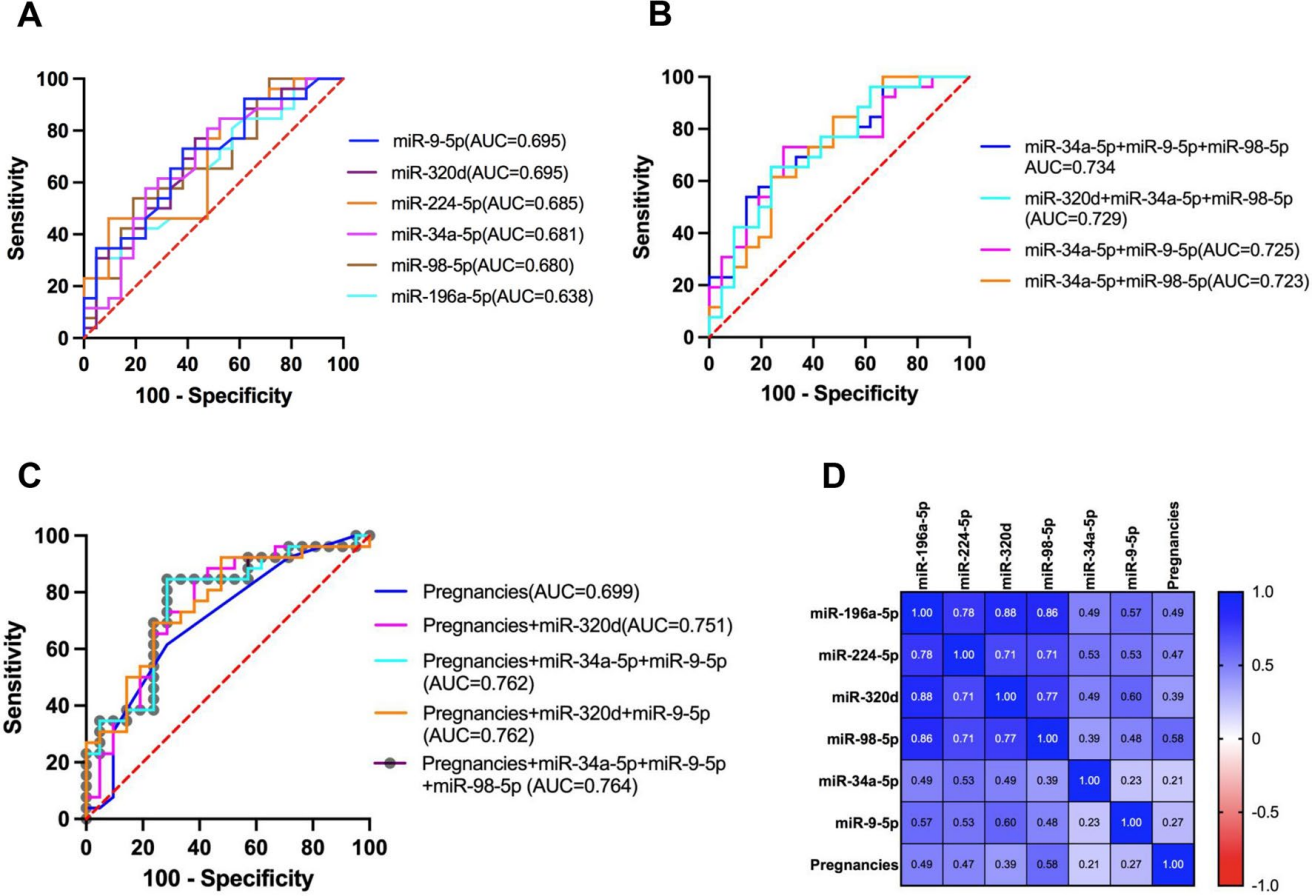
Receiver operating characteristic (ROC) curves for the validated exosomal miRNAs and their combinations. **A** ROC curve of every single exosomal miRNA. **B** ROC curve for combinations of exosomal miRNAs. **C** ROC curve for combinations of the number of pregnancies and exosomal miRNAs. **D** The Pearson correlation coefficient between the number of pregnancies and the relative level of exosomal miRNA expression

We assessed the diagnostic efficacy of each validated exosomal miRNA by simulating the AUC curve. The results were described in (Fig. 5A, Table 4). Among the six miRNAs, miR-9-5p and miR-320d showed the highest diagnostic performance in discriminating PMOP patients and control person, with AUC at 0.695 (95% CI 0.5443–0.8458, sensitivity = 73.08%, specificity = 61.90%) and AUC at 0.695 (95% CI 0.5426–0.8476, sensitivity = 76.92%, specificity = 57.14%), followed by miR-224-5p at AUC = 0.685 (95% CI 0.5302–0.8397, sensitivity = 84.62%, specificity = 47.62%), miR-34a-5p at AUC = 0.681 (95% CI 0.5245–0.8381, sensitivity = 80.77%, specificity = 52.38%) and miR-98-5p at AUC = 0.680 (95% CI 0.5245–0.8345, sensitivity = 100.00%, specificity = 28.57%).

**Table 4:** Diagnostic efficiencies of plasma exosomal miRNA

miRNA	n	AUC	95% CI	*p* value	Cut-off	Sensitivity (%)	Specificity (%)
hsa-miR-9-5p	47	0.6951	0.5443–0.8458	0.0227	0.4789	73.08	61.90
hsa-miR-320d	47	0.6951	0.5426–0.8476	0.0227	0.4860	76.92	57.14
hsa-miR-224-5p	47	0.6850	0.5302–0.8397	0.0307	0.4460	84.62	47.62
hsa-miR-34a-5p	47	0.6813	0.5245–0.8381	0.0341	0.4584	80.77	52.38
hsa-miR-98-5p	47	0.6795	0.5245–0.8345	0.036	0.4177	100.00	28.57
hsa-miR-196a-5p	47	0.6383	0.4783–0.7982	0.1062	0.4603	80.77	42.86
miR-34a-5p + miR-9-5p	47	0.7253	0.5800–0.8705	0.0085	0.4754	73.08	71.43
miR-34a-5p + miR-98-5p	47	0.7234	0.5744–0.8725	0.009	0.3793	100.00	33.33
miR-34a-5p + miR-9-5p + miR-98-5p	47	0.7344	0.5908–0.8780	0.0062	0.5179	65.38	76.19
miR-320d + miR-34a-5p + miR-98-5p	47	0.7289	0.5826–0.8753	0.0075	0.3967	96.15	38.10
Pregnancies	47	0.6987	0.5449–0.8526	0.0202	0.5450	61.54	71.43
Pregnancies + miR-320d	47	0.7509	0.6055–0.8963	0.0034	0.4285	88.46	57.14
Pregnancies + miR-34a-5p + miR-9-5p	47	0.7619	0.6207–0.9031	0.0022	0.4310	84.62	71.43
Pregnancies + miR-320d + miR-9-5p	47	0.7601	0.6210–0.8992	0.0024	0.4048	92.31	52.38
Pregnancies + miR-34a-5p + miR-9-5p + miR-98-5p	47	0.7637	0.6229–0.9045	0.0021	0.4299	84.62	71.43

We further explored whether multiple exosomal miRNA combinations could be used to improve the diagnostic capacity of discriminating PMOP patients. When two exosomal miRNAs were combined, miR-34a-5p + miR-9-5p (AUC = 0.725) and mi-34a-5p + miR-98-5p (AUC = 0.723) were the two best combinations. MiR-34a-5p + miR-9-5p + miR-98-5p (AUC = 0.734) and miR-320d + miR-34a-5p + miR-98-5p (AUC = 0.729) performed best when the three exosomal miRNAs were combined (Fig. 5B, Table 4). The miR-320d + miR-34a-5p + miR-98-5p combination exhibited high sensitivity (96.15%) with a specificity of 38.10%. The addition of more than three exosomal miRNAs does not significantly improve the performance of PMOP detection.

In the section above, we found that the number of pregnancies (PNs) is a risk factor for PMOP, so we combined it with validated exosomal miRNAs. Interestingly, the number of pregnancies could effectively enhance the efficacy of exosomal miRNA for PMOP diagnosis (Fig. 5C, Table 4). First, the number of pregnancies as a single predictor had an AUC of 0.699. When PNs was combined with a single exosomal miRNA, miR-320d is the best choice, with an AUC of 0.751; when it was combined with two exosomal miRNAs, miR-34a-5p + miR-9-5p (AUC = 0.762, sensitivity = 84.62%, specificity = 71.43%) and 320d + miR-9-5p (AUC = 0.760, sensitivity = 92.31%, specificity = 52.38%) show better performance. The best combination of three exosomal miRNAs and PNs is miR-34a-5p + miR-9-5p + miR-98-5p (AUC = 0.764), which was consistent with the exosomal miRNAs training results alone. Pearson correlation analysis of the PNs with exosomal miRNA expression levels showed that the PNs correlated with exosomal miRNA expression was low (< 0.6) (Fig. 5D), indicating that the number of pregnancies (PNs) is an independent predictor that can improve the performance of plasma exosomal miRNAs in diagnosing PMOP.

## Discussion

Here, we identified 27 PMOP-related plasma exosomal miRNAs by next-generation sequencing, and 12 candidate exosomal miRNAs were selected for qRT-PCR validation in another independent cohort, of which six exosomal miRNAs (miR-196-5p, miR-224-5p, miR320d, miR-34a-5p, miR-9-5p, and miR-98-5p) were confirmed to be significantly associated with PMOP. At the same time, we evaluated the performance of exosomal miRNAs in the diagnosis of PMOP for the first time, and the combination of miR-34a-5p + miR-9-5p + miR-98-5p had a better predictive performance with an AUC = 0.734. In addition, the number of pregnancies was found to be an independent risk factor that can improve the performance of exosomal miRNAs in diagnosing PMOP, increasing AUC from 0.734 to 0.764.

In this study, the sequencing cohort included patients with osteoporosis and accompanying fractures, while the control group consisted of healthy individuals of the same age with normal bone density. The validation cohort was composed of elderly women over 65 years of age from the natural community, which represented a real-world situation. The SEC method was used for the isolation of plasma exosomes, which was a more reproduced approach than ultracentrifugation (UC) [30]. Previous studies comparing the applicability of SEC, UC, exoEasy, and exoQuick in plasma exosomal RNomics study showed that SEC had the minimum miRNA binding protein AGO2 and the highest amounts of exosome-specific miRNA, but the lowest nonspecific miRNA [31]. It demonstrated that SEC was a more suitable method for plasma exosomal miRNA research. Then, exosome identification is performed strictly following the guidelines for MISEV2018 [6]. At the sequencing data level, each sample exceeded the minimum number of saturated reads (2.5 M) for plasma exosome miRNA sequencing previously reported in the literature, and the average number of miRNAs identified per sample was close to the number of saturated miRNAs reported in the study of 400 [28]. These details demonstrated that this study is based on high data quality.

A comparison of the sequencing-identified differential expression exosomal miRNAs (DEMs) with the other three DEM datasets revealed that nearly 22% (6/27) of the DEMs were reported in previous studies, indicating the credibility of this study. However, there are a large number of non-intersecting DEMs in different studies. We infer that this is mainly due to different sample collection formats and exosome isolation methods. Studies have reported that when collecting serum, platelets release a large number of exosomes during blood agglutination, thereby diluting the abundance of exosomes in the blood, so plasma can better feedback the real exosomes in human blood than serum collection [32]. Another important reason was that in addition to miRNAs carried in exosomes, there were also a large number of free miRNAs and miRNAs bound to proteins such as AGO2, LDL/HDL, and different isolation methods for exosomes may retain different types of non-exosome miRNAs, thus reflecting different miRNA profile [31, 33]. Therefore, as a blood exosomal biomarker research, it was very important to strictly control the sample collection form and exosome isolation and other test methods; here, we chose plasma collection and SEC isolation exosomes as much as possible to feedback on the real situation of exosomal miRNA in the blood and reduce the non-exosomes-derived miRNA interference.

When we selected miRNAs for validation, we not only considered the results of our sequencing screening, but also included miRNAs that intersected in at least two datasets. At last, six miRNAs were validated by qPCR to prove to be PMOP-associated plasma exosomal miRNAs, including miR-196-5p, miR-224-5p, miR-320d, miR-34a-5p, miR-9-5p, and miR-98-5p. Among them, the upregulation of miR-225-5p has been shown to inhibit osteoblast differentiation by increasing the expression of Pai-1 in the lumbar spine of a rat model of congenital kyphoscoliosis [34]. Muscle-derived miR-34a-5p increased with age in circulating extracellular vesicles and induced senescence of bone marrow stem cells in mice [35]. And the overexpression of miR-9-5p inhibited osteogenic differentiation of bone marrow mesenchymal stem cells by targeting DDX17 [36]. miR-98-5p had been reported to prevent bone regeneration by targeting HMGA2 [37], and the inhibition of miR-98-5p expression could activate PI3K/AKT/GSK3β signaling to promote preosteoblast viability and differentiation by targeting BMP2 [38]. These mechanistic reports support the potential of miR-225-5p, miR-34a-5p, miR-9-5p, and miR-98-5p as diagnostic biomarkers for osteoporosis. The association of miR-196-5p and miR-320d with osteoporosis had not been elaborated, but the MIR196A2 gene expressing miR-196-5p had been reported to be associated with bone mineral density and fracture risk classification in GWAS studies [39]. Even though some of those miRNAs were reported to be associated with bone development and osteoblast differentiation, we were the first to explore and evaluate them as circulating exosomal biomarkers for diagnosing PMOP.

The diagnostic performance evaluation showed that miR-9-5p and miR-320d had the best performance in the single exosomal miRNA model with an AUC = 0.695, miR-34a-5p + miR-9-5p performed best when the two exosomal miRNAs were combined (AUC = 0.725), mi andR-34a-5p + miR-9-5p + miR-98-5p (AUC = 0.734) and miR-320d + miR-34a-5p + miR-98-5p (AUC = 0.729) were the best combinations of the three markers. The miR-320d + miR-34a-5p + miR-98-5p combination exhibited high sensitivity (96.15%) with a specificity of 38.10%. Notably, this study was the first report to evaluate the performance of exosomal miRNAs in diagnosing PMOP. The number of pregnancies was found to be a risk factor for PMOP. Several previous studies have supported the number of pregnancies as a risk factor for PMOP, the conclusion supported by multiple previous studies [21, 22]. However, we were the first study to reveal that the number of pregnancies combined with exosomal miRNA could improve the predictive performance of exosomal miRNA for PMOP.

There were still many limitations to this study that needed to be clarified. Although the size exclusion chromatography method could remove most of the free protein-bound miRNAs in plasma, some miRNAs bound to LDLs would be co-isolated because some LDLs were similar in size to exosomes [40]; therefore, the interference of free miRNAs in plasma on the results could not be completely excluded. The second limitation was that the patients in the sequencing cohort were all patients with osteoporotic fractures, while the validation cohort came from the natural population of the community, which may result in some of the differential exosomal miRNAs screened by sequencing not being validated in the validation cohort; at the same time, the sample size of the validation cohort was relatively small, which may lead to the overfitting of performance evaluation. Finally, although there were differences in AUC values between different diagnostic models, the differences between different models did not pass the Delong test (Additional file 6) [41], which did not indicate significant differences in the diagnostic performance of different models, which might require further validation of larger samples.

## Conclusion

In this study, six PMOP-associated plasma exosomal miRNAs (miR-196-5p, miR-224-5p, miR-320d, miR-34a-5p, miR-9-5p, and miR-98-5p) were identified and validated, and their performance in identifying PMOP was initially evaluated. It was also revealed that the number of pregnancies as an independent risk factor for PMOP could enhance the performance of these exosomal miRNAs in diagnosing PMOP. Further validation of large cohorts is required to confirm the performance of these exosomal miRNAs for the diagnosis of PMOP. What tissues and cells release these exosomal miRNAs also need to be further revealed.

## Supplementary Information

**Additional file 1**. The sequence of primers and probes for candidate exosomal miRNAs.

**Additional file 2**. The number of pregnancies in both cohorts.

**Additional file 3**. The Supplementary Figures of this article. **Fig. S1.** The clean reads number of every sequencing sample, after removing the low-quality reads and the sequences smaller than 18 nt or longer than 32 nt. **Fig. S2.** Relative expression levels (calculated by 2^-ΔΔct^) of plasma exosomal miRNA candidates in the validation set. The comparisons of the two groups were based on an unpaired Student’s t-test, with p < 0.05 as the significance threshold. This figure shows miRNAs without significant differences. (A) miR-152-3p, (B) miR-220b-3p, (C) miR-320c, (D) miR-425-5p, (E) miR-451a, (F) miR-484.

**Additional file 4**. The differential expression exosomal miRNAs of three published datasets.

**Additional file 5**. The target gene list of differential expression exosomal miRNAs in sequencing data.

**Additional file 6**. The p-value of Delong test between different diagnostic models.

## Data Availability

The sequencing data of 24 samples had been uploaded to the SRA database (BioProject: PRJNA951192).
