# Supplementary material for: Identification and evaluation of circulating exosomal miRNAs for the diagnosis of postmenopausal osteoporosis
Source: J Orthop Surg Res. 2023 Jul 26;18:533. doi: 10.1186/s13018-023-04020-z (PMC10373377; doi:10.1186/s13018-023-04020-z)
Supplement: Supplementary file 3 — Additional file 3. The Supplementary Figures of this article. Fig. S1. The clean reads number of every sequencing sample, after removing the low-quality reads and the sequences smaller than 18 nt or longer than 32 nt. Fig. S2. Relative expression levels (calculated by 2-ΔΔct) of plasma exosomal miRNA candidates in the validation set. The comparisons of the two groups were based on an unpaired Student’s t-test, with p < 0.05 as the significance threshold. This figure shows miRNAs without significant differences. (A) miR-152-3p, (B) miR-220b-3p, (C) miR-320c, (D) miR-425-5p, (E) miR-451a, (F) miR-484. [file 13018_2023_4020_MOESM3_ESM.docx]

Supplementary Material

Identification and evaluation of circulating exosomal miRNAs for the diagnosis of Postmenopausal osteoporosis

Zhibang Sun^1^†, Junjie Shi^1^†, Chenyang Yang^1^†, Xukun Chen^1^, Jiaqi Chu^1^, Jing Chen^2^, Yuan Wang^2^, Chenxin Zhu^1^, Jinze Xu^1^, Guozhen Tang^1^, and Song Shao^1^*

*** Correspondence:** Song Shao: [1255319122@qq.com](mailto:1255319122@qq.com)

# Supplementary Figures and Tables

## Supplementary Figures
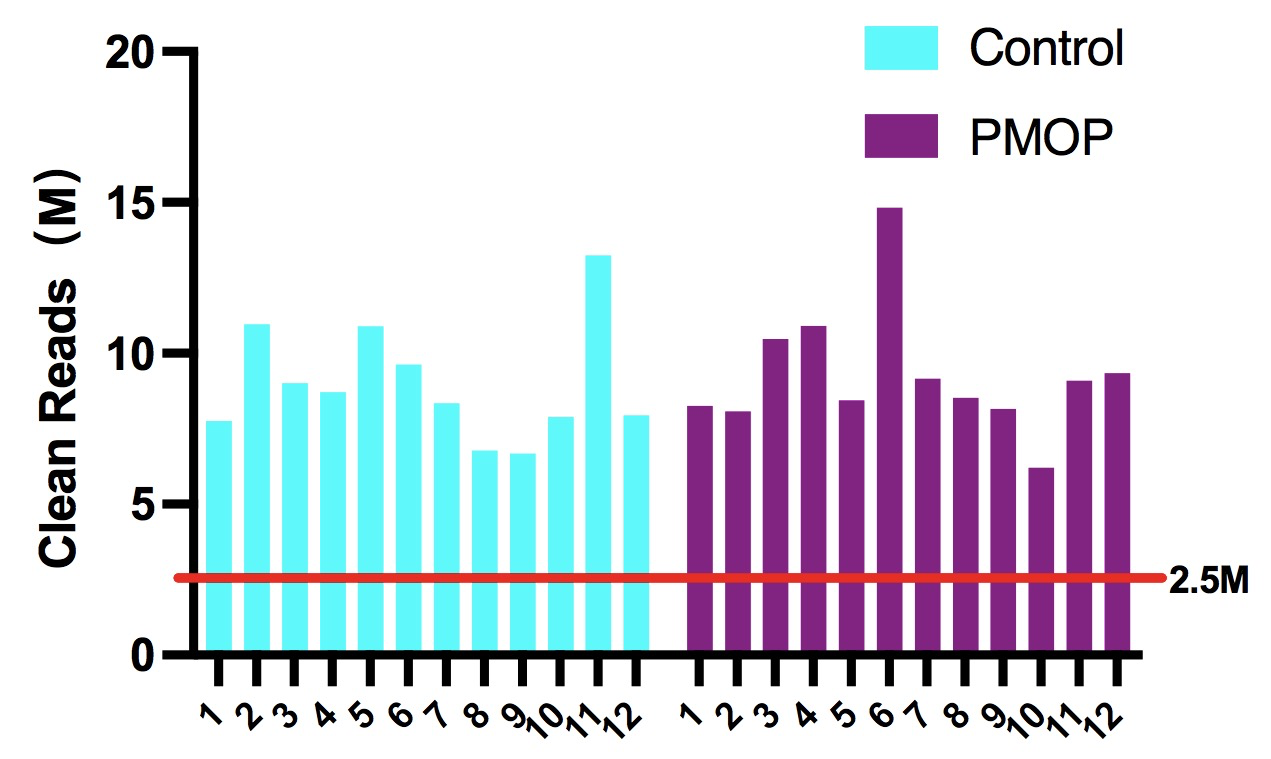
Supplementary Figure S1. The clean reads number of every sequencing sample, after removing the low-quality reads and the sequences smaller than 18 nt or longer than 32 nt.
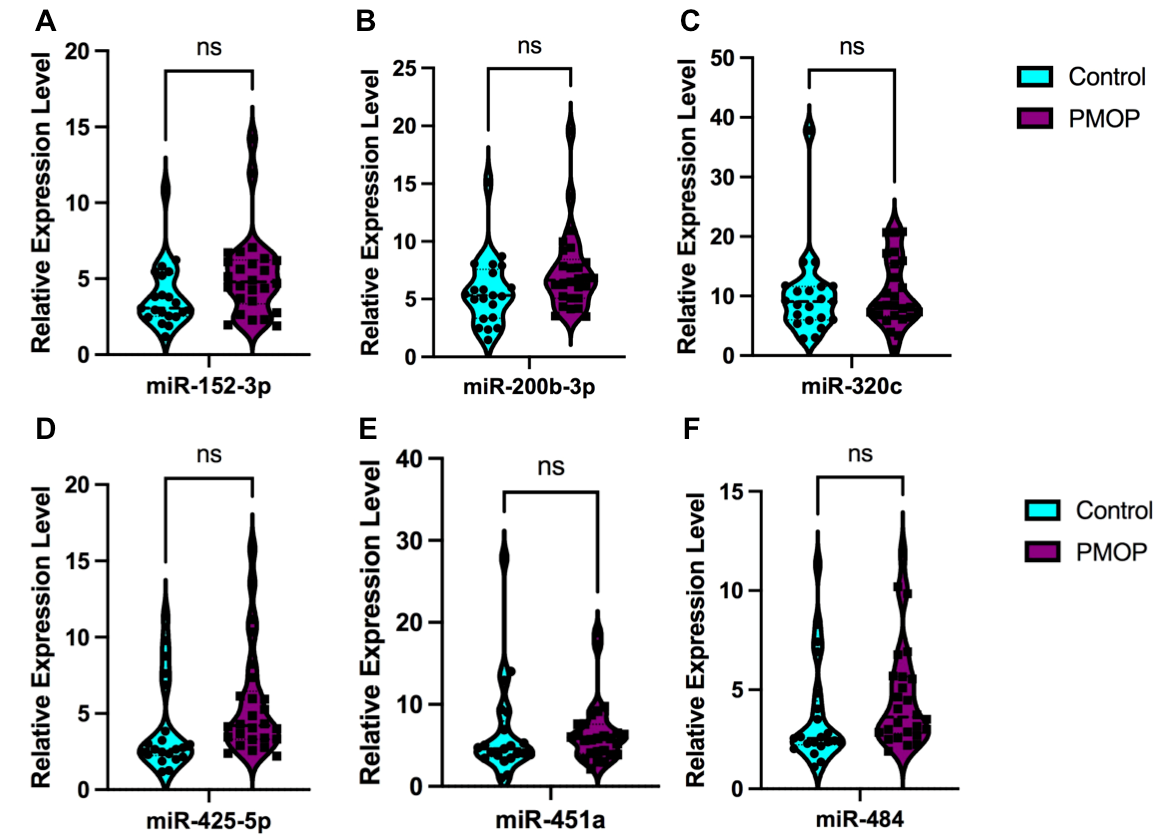
Supplementary Figure S2. Relative expression levels (calculated by 2^-ΔΔct^) of plasma exosomal miRNA candidates in the validation set. The comparisons of the two groups were based on an unpaired Student’s t-test, with p < 0.05 as the significance threshold. This figure shows miRNAs without significant differences. (A) miR-152-3p, (B) miR-220b-3p, (C) miR-320c, (D) miR-425-5p, (E) miR-451a, (F) miR-484.

## Supplementary Tables

**Additional file 1.** The sequence of primers and probes for candidate exosomal miRNAs.

**Additional file 2.**  The number of pregnancies in both cohorts.

**Additional file 4.** The differential expression exosomal miRNAs of three published datasets.

**Additional file 5.** The target gene list of differential expression exosomal miRNAs in sequencing data.

**Additional file 6.** The p-value of Delong test between different diagnostic models.
